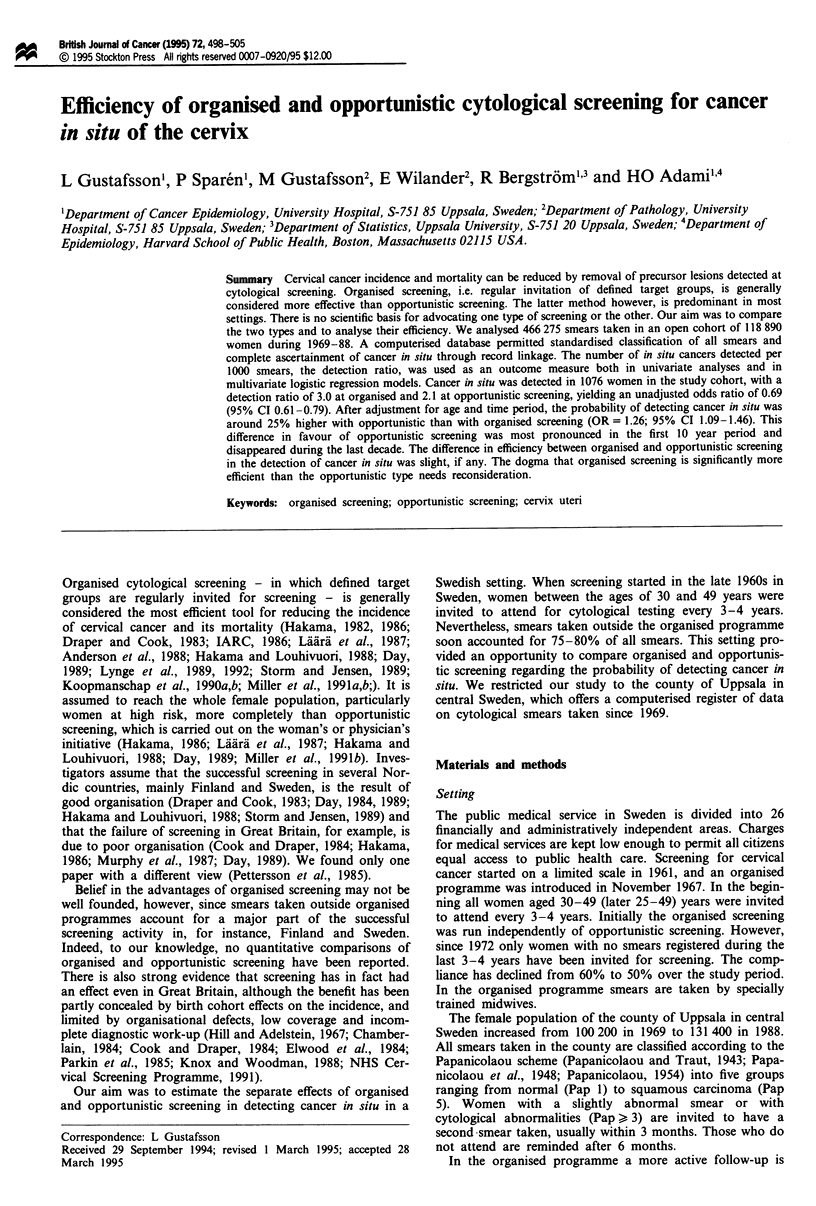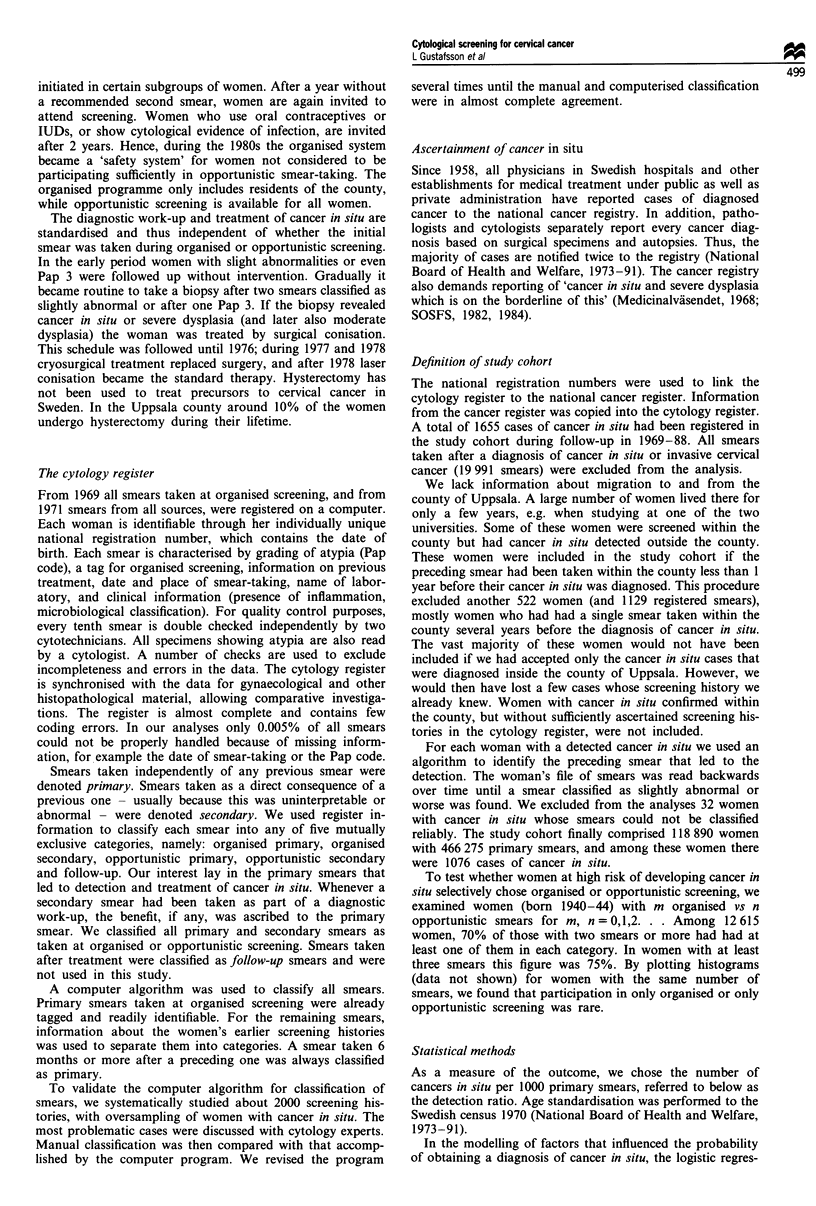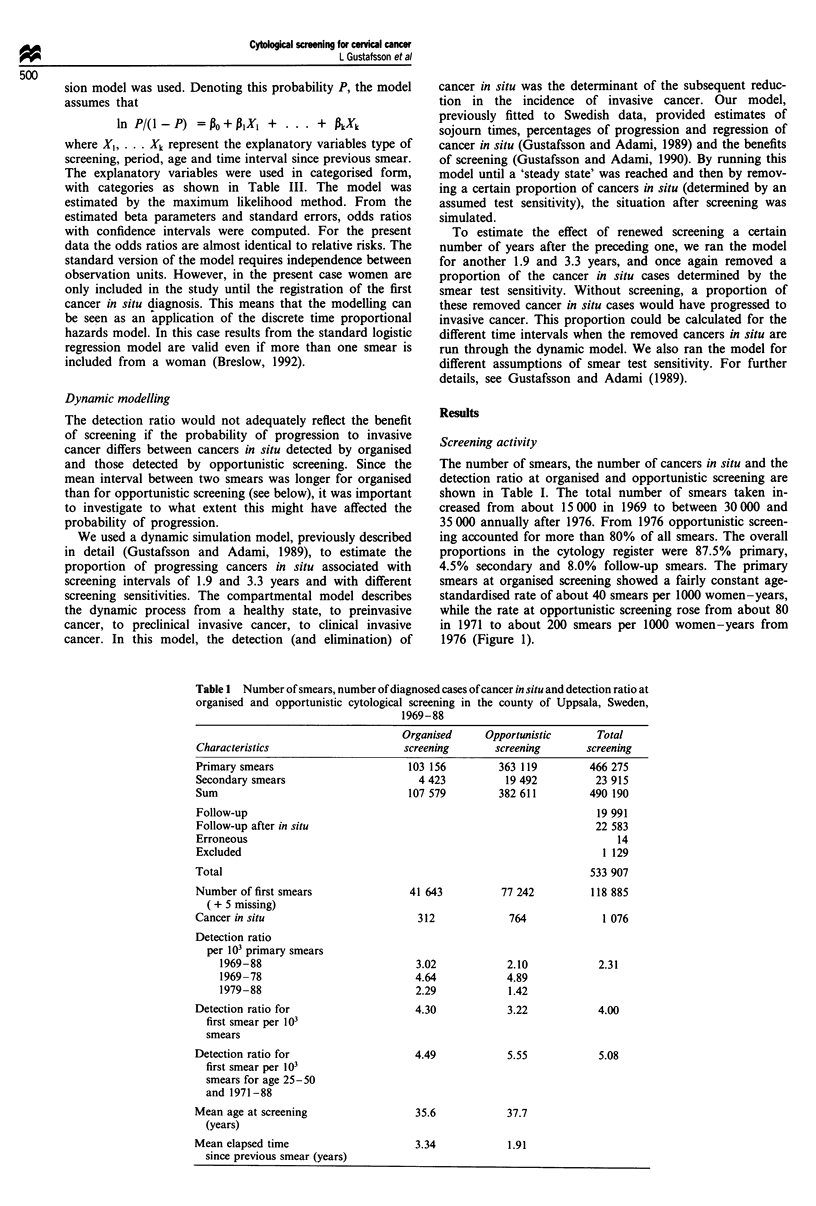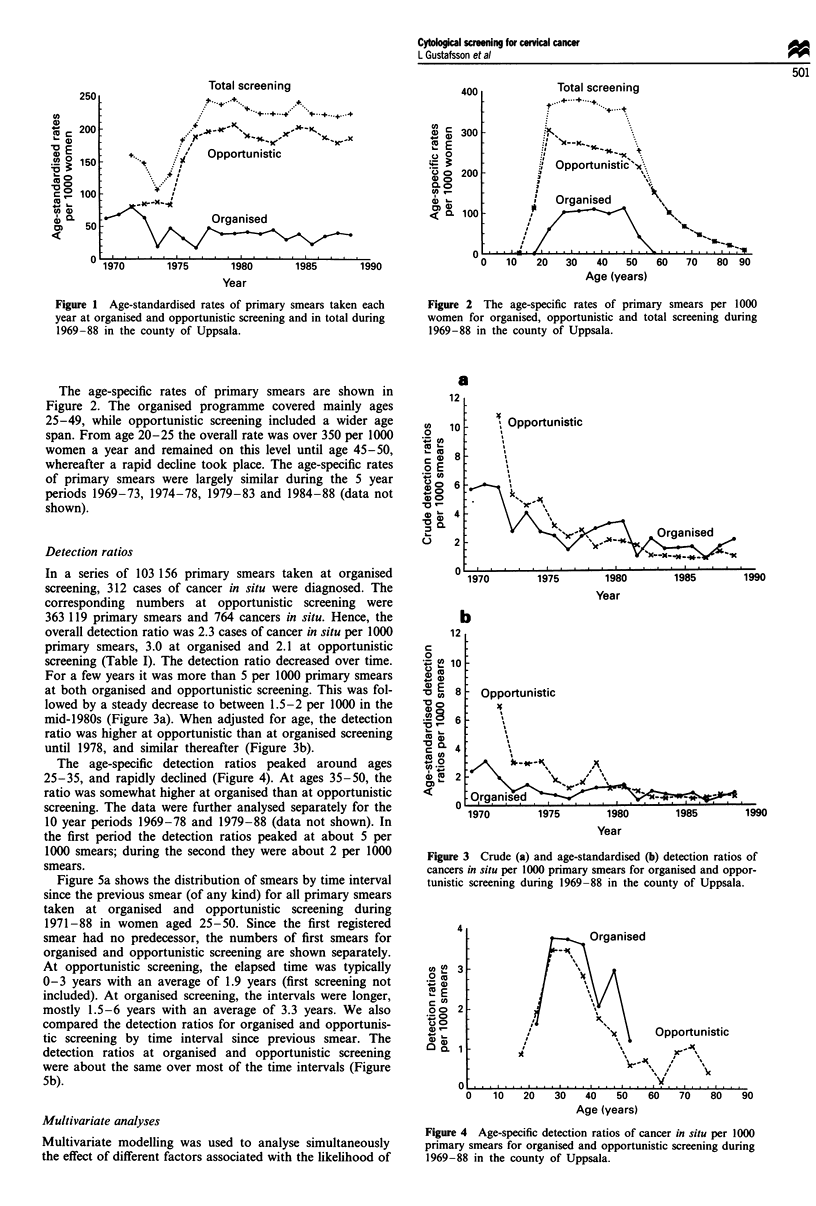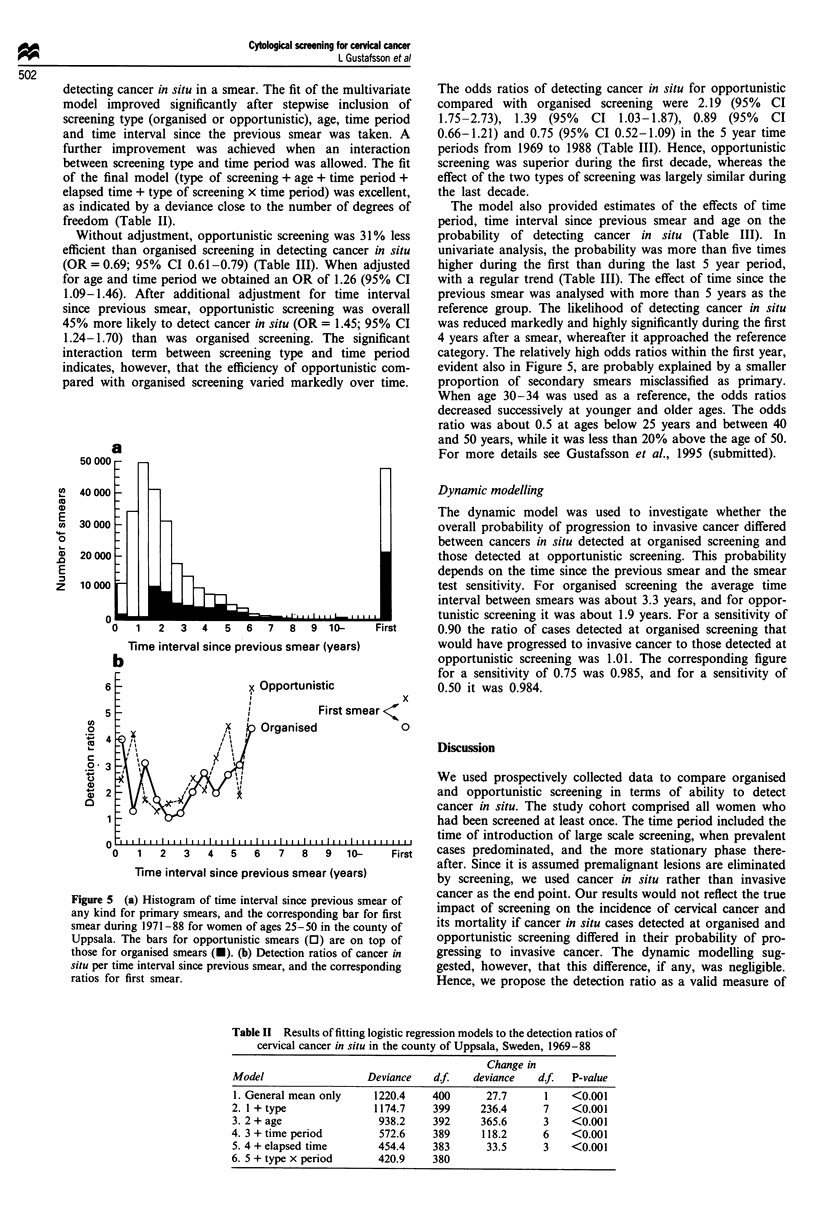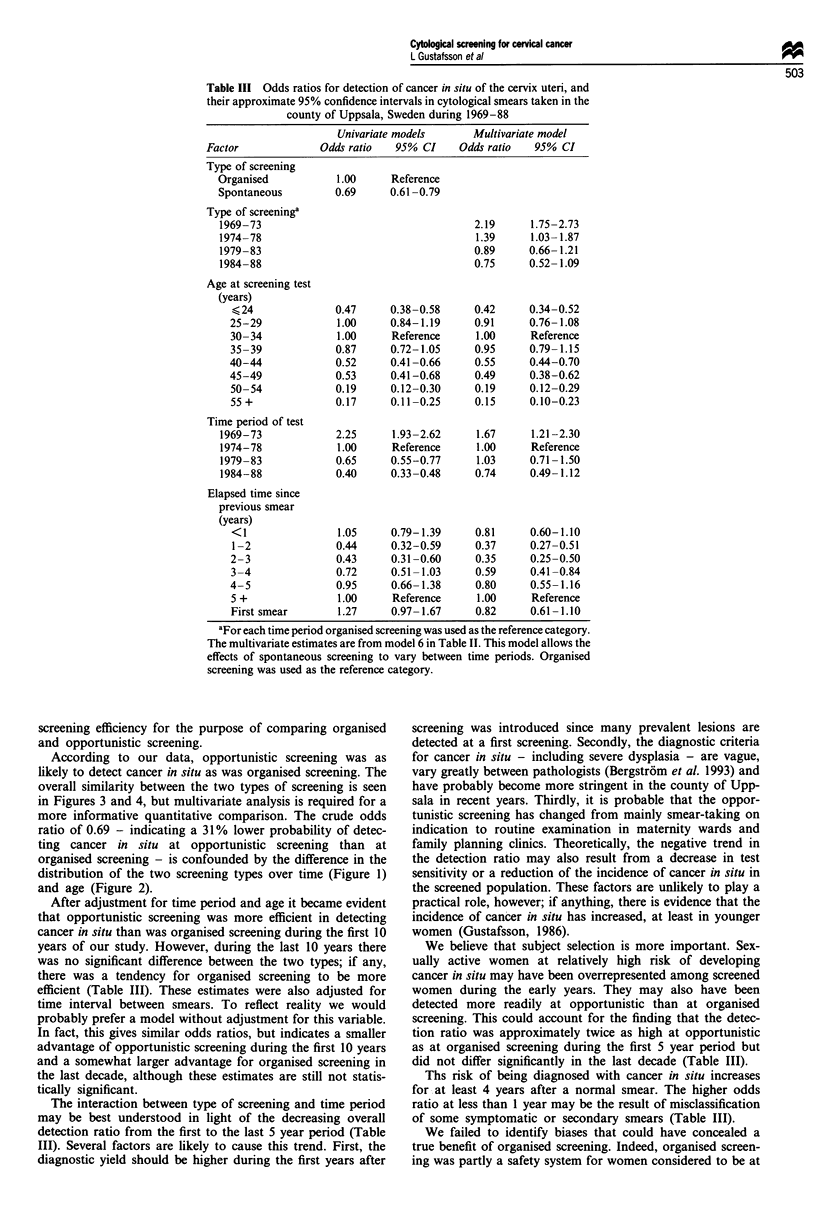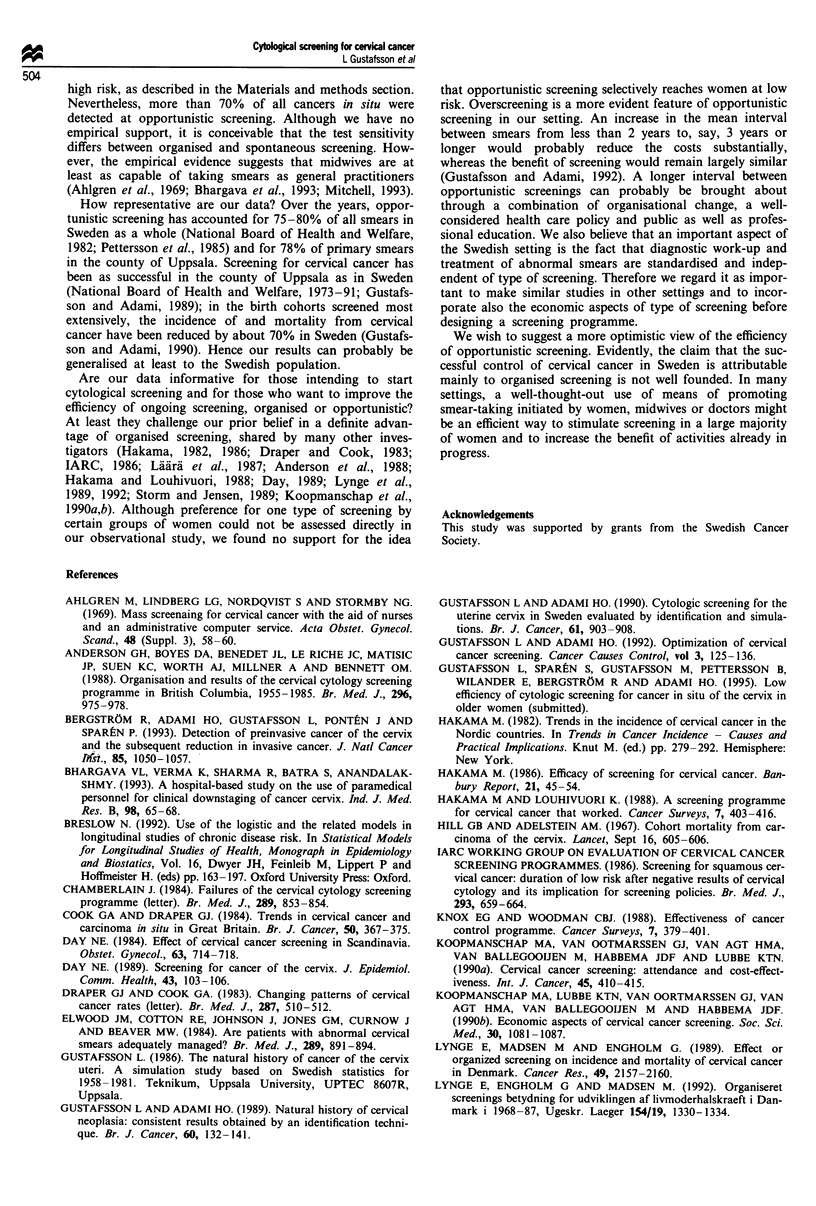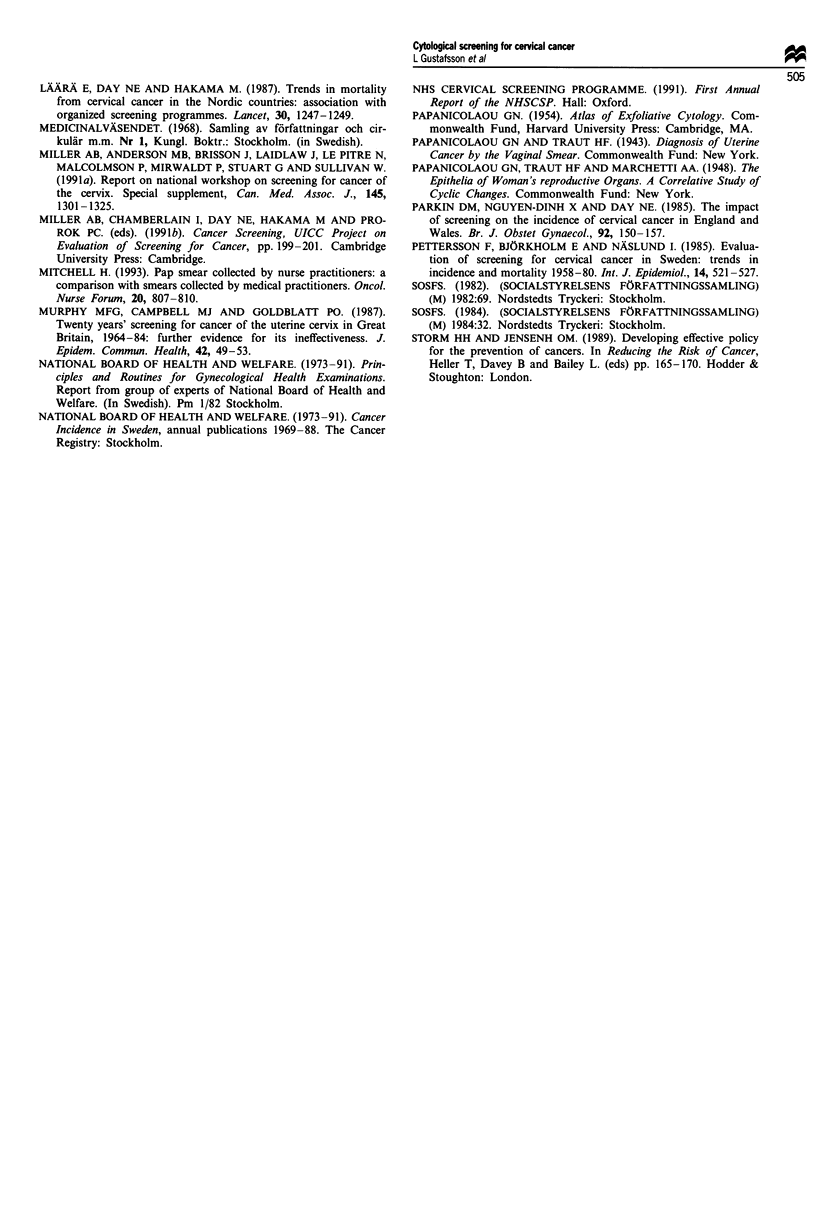# Efficiency of organised and opportunistic cytological screening for cancer in situ of the cervix.

**DOI:** 10.1038/bjc.1995.362

**Published:** 1995-08

**Authors:** L. Gustafsson, P. Sparén, M. Gustafsson, E. Wilander, R. Bergström, H. O. Adami

**Affiliations:** Department of Cancer Epidemiology, University Hospital, Uppsala, Sweden.

## Abstract

Cervical cancer incidence and mortality can be reduced by removal of precursor lesions detected at cytological screening. Organised screening, i.e. regular invitation of defined target groups, is generally considered more effective than opportunistic screening. The latter method however, is predominant in most settings. There is no scientific basis for advocating one type of screening or the other. Our aim was to compare the two types and to analyse their efficiency. We analysed 466,275 smears taken in an open cohort of 118,890 women during 1969-88. A computerised database permitted standardised classification of all smears and complete ascertainment of cancer in situ through record linkage. The number of in situ cancers detected per 1000 smears, the detection ratio, was used as an outcome measure both in univariate analyses and in multivariate logistic regression models. Cancer in situ was detected in 1076 women in the study cohort, with a detection ratio of 3.0 at organised and 2.1 at opportunistic screening, yielding an unadjusted odds ratio of 0.69 (95% CI 0.61-0.79). After adjustment for age and time period, the probability of detecting cancer in situ was around 25% higher with opportunistic than with organised screening (OR = 1.26; 95% CI 1.09-1.46). This difference in favour of opportunistic screening was most pronounced in the first 10 year period and disappeared during the last decade. The difference in efficiency between organised and opportunistic screening in the detection of cancer in situ was slight, if any. The dogma that organised screening is significantly more efficient than the opportunistic type needs reconsideration.


British Journal of Cancer (1995) 72, 498-505
© 1995 Stockton Press All rights reserved 0007-0920/95 $12.00

### Efficiency of organised and opportunistic cytological screening for cancer *in situ* of the cervix

L Gustafsson[1], P Sparén[1], M Gustafsson[2], E Wilander[2], R Bergström[1,3] and HO Adami[1,4]

[1]*Department of Cancer Epidemiology, University Hospital, S-751 85 Uppsala, Sweden;* [2]*Department of Pathology, University Hospital, S-751 85 Uppsala, Sweden;* [3]*Department of Statistics, Uppsala University, S-751 20 Uppsala, Sweden;* [4]*Department of Epidemiology, Harvard School of Public Health, Boston, Massachusetts 02115 USA.*

**Summary** Cervical cancer incidence and mortality can be reduced by removal of precursor lesions detected at cytological screening. Organised screening, i.e. regular invitation of defined target groups, is generally considered more effective than opportunistic screening. The latter method however, is predominant in most settings. There is no scientific basis for advocating one type of screening or the other. Our aim was to compare the two types and to analyse their efficiency. We analysed 466 275 smears taken in an open cohort of 118 890 women during 1969-88. A computerised database permitted standardised classification of all smears and complete ascertainment of cancer *in situ* through record linkage. The number of *in situ* cancers detected per 1000 smears, the detection ratio, was used as an outcome measure both in univariate analyses and in multivariate logistic regression models. Cancer *in situ* was detected in 1076 women in the study cohort, with a detection ratio of 3.0 at organised and 2.1 at opportunistic screening, yielding an unadjusted odds ratio of 0.69 (95% CI 0.61-0.79). After adjustment for age and time period, the probability of detecting cancer *in situ* was around 25% higher with opportunistic than with organised screening (OR = 1.26; 95% CI 1.09-1.46). This difference in favour of opportunistic screening was most pronounced in the first 10 year period and disappeared during the last decade. The difference in efficiency between organised and opportunistic screening in the detection of cancer *in situ* was slight, if any. The dogma that organised screening is significantly more efficient than the opportunistic type needs reconsideration.

**Keywords:** organised screening; opportunistic screening; cervix uteri

Organised cytological screening - in which defined target groups are regularly invited for screening - is generally considered the most efficient tool for reducing the incidence of cervical cancer and its mortality (Hakama, 1982, 1986; Draper and Cook, 1983; IARC, 1986; Läärä *et al.*, 1987; Anderson *et al.*, 1988; Hakama and Louhivuori, 1988; Day, 1989; Lynge *et al.*, 1989, 1992; Storm and Jensen, 1989; Koopmanschap *et al.*, 1990*a*,*b*; Miller *et al.*, 1991*a*,*b*;). It is assumed to reach the whole female population, particularly women at high risk, more completely than opportunistic screening, which is carried out on the woman's or physician's initiative (Hakama, 1986; Läärä *et al.*, 1987; Hakama and Louhivuori, 1988; Day, 1989; Miller *et al.*, 1991*b*). Investigators assume that the successful screening in several Nordic countries, mainly Finland and Sweden, is the result of good organisation (Draper and Cook, 1983; Day, 1984, 1989; Hakama and Louhivuori, 1988; Storm and Jensen, 1989) and that the failure of screening in Great Britain, for example, is due to poor organisation (Cook and Draper, 1984; Hakama, 1986; Murphy *et al.*, 1987; Day, 1989). We found only one paper with a different view (Pettersson *et al.*, 1985).

Belief in the advantages of organised screening may not be well founded, however, since smears taken outside organised programmes account for a major part of the successful screening activity in, for instance, Finland and Sweden. Indeed, to our knowledge, no quantitative comparisons of organised and opportunistic screening have been reported. There is also strong evidence that screening has in fact had an effect even in Great Britain, although the benefit has been partly concealed by birth cohort effects on the incidence, and limited by organisational defects, low coverage and incomplete diagnostic work-up (Hill and Adelstein, 1967; Chamberlain, 1984; Cook and Draper, 1984; Elwood *et al.*, 1984; Parkin *et al.*, 1985; Knox and Woodman, 1988; NHS Cervical Screening Programme, 1991).

Our aim was to estimate the separate effects of organised and opportunistic screening in detecting cancer *in situ* in a Swedish setting. When screening started in the late 1960s in Sweden, women between the ages of 30 and 49 years were invited to attend for cytological testing every 3-4 years. Nevertheless, smears taken outside the organised programme soon accounted for 75-80% of all smears. This setting provided an opportunity to compare organised and opportunistic screening regarding the probability of detecting cancer *in situ*. We restricted our study to the county of Uppsala in central Sweden, which offers a computerised register of data on cytological smears taken since 1969.

Correspondence: L Gustafsson
Received 29 September 1994; revised 1 March 1995; accepted 28 March 1995

### Materials and methods

### *Setting*

The public medical service in Sweden is divided into 26 financially and administratively independent areas. Charges for medical services are kept low enough to permit all citizens equal access to public health care. Screening for cervical cancer started on a limited scale in 1961, and an organised programme was introduced in November 1967. In the beginning all women aged 30-49 (later 25-49) years were invited to attend every 3-4 years. Initially the organised screening was run independently of opportunistic screening. However, since 1972 only women with no smears registered during the last 3-4 years have been invited for screening. The compliance has declined from 60% to 50% over the study period. In the organised programme smears are taken by specially trained midwives.

The female population of the county of Uppsala in central Sweden increased from 100 200 in 1969 to 131 400 in 1988. All smears taken in the county are classified according to the Papanicolaou scheme (Papanicolaou and Traut, 1943; Papanicolaou *et al.*, 1948; Papanicolaou, 1954) into five groups ranging from normal (Pap 1) to squamous carcinoma (Pap 5). Women with a slightly abnormal smear or with cytological abnormalities (Pap ⩾ 3) are invited to have a second smear taken, usually within 3 months. Those who do not attend are reminded after 6 months.

In the organised programme a more active follow-up is initiated in certain subgroups of women. After a year without a recommended second smear, women are again invited to attend screening. Women who use oral contraceptives or IUDs, or show cytological evidence of infection, are invited after 2 years. Hence, during the 1980s the organised system became a 'safety system' for women not considered to be participating sufficiently in opportunistic smear-taking. The organised programme only includes residents of the county, while opportunistic screening is available for all women.

The diagnostic work-up and treatment of cancer *in situ* are standardised and thus independent of whether the initial smear was taken during organised or opportunistic screening. In the early period women with slight abnormalities or even Pap 3 were followed up without intervention. Gradually it became routine to take a biopsy after two smears classified as slightly abnormal or after one Pap 3. If the biopsy revealed cancer *in situ* or severe dysplasia (and later also moderate dysplasia) the woman was treated by surgical conisation. This schedule was followed until 1976; during 1977 and 1978 cryosurgical treatment replaced surgery, and after 1978 laser conisation became the standard therapy. Hysterectomy has not been used to treat precursors to cervical cancer in Sweden. In the Uppsala county around 10% of the women undergo hysterectomy during their lifetime.

### *The cytology register*

From 1969 all smears taken at organised screening, and from 1971 smears from all sources, were registered on a computer. Each woman is identifiable through her individually unique national registration number, which contains the date of birth. Each smear is characterised by grading of atypia (Pap code), a tag for organised screening, information on previous treatment, date and place of smear-taking, name of laboratory, and clinical information (presence of inflammation, microbiological classification). For quality control purposes, every tenth smear is double checked independently by two cytotechnicians. All specimens showing atypia are also read by a cytologist. A number of checks are used to exclude incompleteness and errors in the data. The cytology register is synchronised with the data for gynaecological and other histopathological material, allowing comparative investigations. The register is almost complete and contains few coding errors. In our analyses only 0.005% of all smears could not be properly handled because of missing information, for example the date of smear-taking or the Pap code.

Smears taken independently of any previous smear were denoted *primary*. Smears taken as a direct consequence of a previous one – usually because this was uninterpretable or abnormal – were denoted *secondary*. We used register information to classify each smear into any of five mutually exclusive categories, namely: organised primary, organised secondary, opportunistic primary, opportunistic secondary and follow-up. Our interest lay in the primary smears that led to detection and treatment of cancer *in situ*. Whenever a secondary smear had been taken as part of a diagnostic work-up, the benefit, if any, was ascribed to the primary smear. We classified all primary and secondary smears as taken at organised or opportunistic screening. Smears taken after treatment were classified as *follow-up* smears and were not used in this study.

A computer algorithm was used to classify all smears. Primary smears taken at organised screening were already tagged and readily identifiable. For the remaining smears, information about the women's earlier screening histories was used to separate them into categories. A smear taken 6 months or more after a preceding one was always classified as primary.

To validate the computer algorithm for classification of smears, we systematically studied about 2000 screening histories, with oversampling of women with cancer *in situ*. The most problematic cases were discussed with cytology experts. Manual classification was then compared with that accomplished by the computer program. We revised the program several times until the manual and computerised classification were in almost complete agreement.

### *Ascertainment of cancer* in situ

Since 1958, all physicians in Swedish hospitals and other establishments for medical treatment under public as well as private administration have reported cases of diagnosed cancer to the national cancer registry. In addition, pathologists and cytologists separately report every cancer diagnosis based on surgical specimens and autopsies. Thus, the majority of cases are notified twice to the registry (National Board of Health and Welfare, 1973–91). The cancer registry also demands reporting of 'cancer *in situ* and severe dysplasia which is on the borderline of this' (Medicinalväsendet, 1968; SOSFS, 1982, 1984).

### *Definition of study cohort*

The national registration numbers were used to link the cytology register to the national cancer register. Information from the cancer register was copied into the cytology register. A total of 1655 cases of cancer *in situ* had been registered in the study cohort during follow-up in 1969–88. All smears taken after a diagnosis of cancer *in situ* or invasive cervical cancer (19 991 smears) were excluded from the analysis.

We lack information about migration to and from the county of Uppsala. A large number of women lived there for only a few years, e.g. when studying at one of the two universities. Some of these women were screened within the county but had cancer *in situ* detected outside the county. These women were included in the study cohort if the preceding smear had been taken within the county less than 1 year before their cancer *in situ* was diagnosed. This procedure excluded another 522 women (and 1129 registered smears), mostly women who had had a single smear taken within the county several years before the diagnosis of cancer *in situ*. The vast majority of these women would not have been included if we had accepted only the cancer *in situ* cases that were diagnosed inside the county of Uppsala. However, we would then have lost a few cases whose screening history we already knew. Women with cancer *in situ* confirmed within the county, but without sufficiently ascertained screening histories in the cytology register, were not included.

For each woman with a detected cancer *in situ* we used an algorithm to identify the preceding smear that led to the detection. The woman's file of smears was read backwards over time until a smear classified as slightly abnormal or worse was found. We excluded from the analyses 32 women with cancer *in situ* whose smears could not be classified reliably. The study cohort finally comprised 118 890 women with 466 275 primary smears, and among these women there were 1076 cases of cancer *in situ*.

To test whether women at high risk of developing cancer *in situ* selectively chose organised or opportunistic screening, we examined women (born 1940–44) with $m$ organised *vs* $n$ opportunistic smears for $m$, $n = 0,1,2...$ Among 12 615 women, 70% of those with two smears or more had had at least one of them in each category. In women with at least three smears this figure was 75%. By plotting histograms (data not shown) for women with the same number of smears, we found that participation in only organised or only opportunistic screening was rare.

### *Statistical methods*

As a measure of the outcome, we chose the number of cancers *in situ* per 1000 primary smears, referred to below as the detection ratio. Age standardisation was performed to the Swedish census 1970 (National Board of Health and Welfare, 1973–91).

In the modelling of factors that influenced the probability of obtaining a diagnosis of cancer *in situ*, the logistic regression model was used. Denoting this probability $P$, the model assumes that

$$\ln P/(1-P) = \beta_0 + \beta_1 X_1 + \ldots + \beta_k X_k$$

where $X_1, \ldots X_k$ represent the explanatory variables type of screening, period, age and time interval since previous smear. The explanatory variables were used in categorised form, with categories as shown in Table III. The model was estimated by the maximum likelihood method. From the estimated beta parameters and standard errors, odds ratios with confidence intervals were computed. For the present data the odds ratios are almost identical to relative risks. The standard version of the model requires independence between observation units. However, in the present case women are only included in the study until the registration of the first cancer *in situ* diagnosis. This means that the modelling can be seen as an application of the discrete time proportional hazards model. In this case results from the standard logistic regression model are valid even if more than one smear is included from a woman (Breslow, 1992).

*Dynamic modelling*

The detection ratio would not adequately reflect the benefit of screening if the probability of progression to invasive cancer differs between cancers *in situ* detected by organised and those detected by opportunistic screening. Since the mean interval between two smears was longer for organised than for opportunistic screening (see below), it was important to investigate to what extent this might have affected the probability of progression.

We used a dynamic simulation model, previously described in detail (Gustafsson and Adami, 1989), to estimate the proportion of progressing cancers *in situ* associated with screening intervals of 1.9 and 3.3 years and with different screening sensitivities. The compartmental model describes the dynamic process from a healthy state, to preinvasive cancer, to preclinical invasive cancer, to clinical invasive cancer. In this model, the detection (and elimination) of cancer *in situ* was the determinant of the subsequent reduction in the incidence of invasive cancer. Our model, previously fitted to Swedish data, provided estimates of sojourn times, percentages of progression and regression of cancer *in situ* (Gustafsson and Adami, 1989) and the benefits of screening (Gustafsson and Adami, 1990). By running this model until a 'steady state' was reached and then by removing a certain proportion of cancers *in situ* (determined by an assumed test sensitivity), the situation after screening was simulated.

To estimate the effect of renewed screening a certain number of years after the preceding one, we ran the model for another 1.9 and 3.3 years, and once again removed a proportion of the cancer *in situ* cases determined by the smear test sensitivity. Without screening, a proportion of these removed cancer *in situ* cases would have progressed to invasive cancer. This proportion could be calculated for the different time intervals when the removed cancers *in situ* are run through the dynamic model. We also ran the model for different assumptions of smear test sensitivity. For further details, see Gustafsson and Adami (1989).

### Results

*Screening activity*

The number of smears, the number of cancers *in situ* and the detection ratio at organised and opportunistic screening are shown in Table I. The total number of smears taken increased from about 15 000 in 1969 to between 30 000 and 35 000 annually after 1976. From 1976 opportunistic screening accounted for more than 80% of all smears. The overall proportions in the cytology register were 87.5% primary, 4.5% secondary and 8.0% follow-up smears. The primary smears at organised screening showed a fairly constant age-standardised rate of about 40 smears per 1000 women-years, while the rate at opportunistic screening rose from about 80 in 1971 to about 200 smears per 1000 women-years from 1976 (Figure 1).

**Table I** Number of smears, number of diagnosed cases of cancer *in situ* and detection ratio at organised and opportunistic cytological screening in the county of Uppsala, Sweden, 1969-88

| *Characteristics* | *Organised screening* | *Opportunistic screening* | *Total screening* |
|---|---|---|---|
| Primary smears | 103 156 | 363 119 | 466 275 |
| Secondary smears | 4 423 | 19 492 | 23 915 |
| Sum | 107 579 | 382 611 | 490 190 |
| Follow-up | | | 19 991 |
| Follow-up after *in situ* | | | 22 583 |
| Erroneous | | | 14 |
| Excluded | | | 1 129 |
| Total | | | 533 907 |
| Number of first smears (+5 missing) | 41 643 | 77 242 | 118 885 |
| Cancer *in situ* | 312 | 764 | 1 076 |
| Detection ratio per $10^3$ primary smears | | | |
| 1969-88 | 3.02 | 2.10 | 2.31 |
| 1969-78 | 4.64 | 4.89 | |
| 1979-88 | 2.29 | 1.42 | |
| Detection ratio for first smear per $10^3$ smears | 4.30 | 3.22 | 4.00 |
| Detection ratio for first smear per $10^3$ smears for age 25-50 and 1971-88 | 4.49 | 5.55 | 5.08 |
| Mean age at screening (years) | 35.6 | 37.7 | |
| Mean elapsed time since previous smear (years) | 3.34 | 1.91 | |

**Figure 1** Age-standardised rates of primary smears taken each year at organised and opportunistic screening and in total during 1969–88 in the county of Uppsala.

**Figure 2** The age-specific rates of primary smears per 1000 women for organised, opportunistic and total screening during 1969–88 in the county of Uppsala.

The age-specific rates of primary smears are shown in Figure 2. The organised programme covered mainly ages 25–49, while opportunistic screening included a wider age span. From age 20–25 the overall rate was over 350 per 1000 women a year and remained on this level until age 45–50, whereafter a rapid decline took place. The age-specific rates of primary smears were largely similar during the 5 year periods 1969–73, 1974–78, 1979–83 and 1984–88 (data not shown).

### *Detection ratios*

In a series of 103 156 primary smears taken at organised screening, 312 cases of cancer *in situ* were diagnosed. The corresponding numbers at opportunistic screening were 363 119 primary smears and 764 cancers *in situ*. Hence, the overall detection ratio was 2.3 cases of cancer *in situ* per 1000 primary smears, 3.0 at organised and 2.1 at opportunistic screening (Table I). The detection ratio decreased over time. For a few years it was more than 5 per 1000 primary smears at both organised and opportunistic screening. This was followed by a steady decrease to between 1.5–2 per 1000 in the mid-1980s (Figure 3a). When adjusted for age, the detection ratio was higher at opportunistic than at organised screening until 1978, and similar thereafter (Figure 3b).

The age-specific detection ratios peaked around ages 25–35, and rapidly declined (Figure 4). At ages 35–50, the ratio was somewhat higher at organised than at opportunistic screening. The data were further analysed separately for the 10 year periods 1969–78 and 1979–88 (data not shown). In the first period the detection ratios peaked at about 5 per 1000 smears; during the second they were about 2 per 1000 smears.

Figure 5a shows the distribution of smears by time interval since the previous smear (of any kind) for all primary smears taken at organised and opportunistic screening during 1971–88 in women aged 25–50. Since the first registered smear had no predecessor, the numbers of first smears for organised and opportunistic screening are shown separately. At opportunistic screening, the elapsed time was typically 0–3 years with an average of 1.9 years (first screening not included). At organised screening, the intervals were longer, mostly 1.5–6 years with an average of 3.3 years. We also compared the detection ratios for organised and opportunistic screening by time interval since previous smear. The detection ratios at organised and opportunistic screening were about the same over most of the time intervals (Figure 5b).

### *Multivariate analyses*

Multivariate modelling was used to analyse simultaneously the effect of different factors associated with the likelihood of

**Figure 3** Crude (**a**) and age-standardised (**b**) detection ratios of cancers *in situ* per 1000 primary smears for organised and opportunistic screening during 1969–88 in the county of Uppsala.

**Figure 4** Age-specific detection ratios of cancer *in situ* per 1000 primary smears for organised and opportunistic screening during 1969–88 in the county of Uppsala.

detecting cancer *in situ* in a smear. The fit of the multivariate model improved significantly after stepwise inclusion of screening type (organised or opportunistic), age, time period and time interval since the previous smear was taken. A further improvement was achieved when an interaction between screening type and time period was allowed. The fit of the final model (type of screening + age + time period + elapsed time + type of screening × time period) was excellent, as indicated by a deviance close to the number of degrees of freedom (Table II).

Without adjustment, opportunistic screening was 31% less efficient than organised screening in detecting cancer *in situ* (OR = 0.69; 95% CI 0.61–0.79) (Table III). When adjusted for age and time period we obtained an OR of 1.26 (95% CI 1.09–1.46). After additional adjustment for time interval since previous smear, opportunistic screening was overall 45% more likely to detect cancer *in situ* (OR = 1.45; 95% CI 1.24–1.70) than was organised screening. The significant interaction term between screening type and time period indicates, however, that the efficiency of opportunistic compared with organised screening varied markedly over time.

**Figure 5** (a) Histogram of time interval since previous smear of any kind for primary smears, and the corresponding bar for first smear during 1971–88 for women of ages 25–50 in the county of Uppsala. The bars for opportunistic smears (□) are on top of those for organised smears (■). (b) Detection ratios of cancer *in situ* per time interval since previous smear, and the corresponding ratios for first smear.

The odds ratios of detecting cancer *in situ* for opportunistic compared with organised screening were 2.19 (95% CI 1.75–2.73), 1.39 (95% CI 1.03–1.87), 0.89 (95% CI 0.66–1.21) and 0.75 (95% CI 0.52–1.09) in the 5 year time periods from 1969 to 1988 (Table III). Hence, opportunistic screening was superior during the first decade, whereas the effect of the two types of screening was largely similar during the last decade.

The model also provided estimates of the effects of time period, time interval since previous smear and age on the probability of detecting cancer *in situ* (Table III). In univariate analysis, the probability was more than five times higher during the first than during the last 5 year period, with a regular trend (Table III). The effect of time since the previous smear was analysed with more than 5 years as the reference group. The likelihood of detecting cancer *in situ* was reduced markedly and highly significantly during the first 4 years after a smear, whereafter it approached the reference category. The relatively high odds ratios within the first year, evident also in Figure 5, are probably explained by a smaller proportion of secondary smears misclassified as primary. When age 30–34 was used as a reference, the odds ratios decreased successively at younger and older ages. The odds ratio was about 0.5 at ages below 25 years and between 40 and 50 years, while it was less than 20% above the age of 50. For more details see Gustafsson *et al.*, 1995 (submitted).

*Dynamic modelling*

The dynamic model was used to investigate whether the overall probability of progression to invasive cancer differed between cancers *in situ* detected at organised screening and those detected at opportunistic screening. This probability depends on the time since the previous smear and the smear test sensitivity. For organised screening the average time interval between smears was about 3.3 years, and for opportunistic screening it was about 1.9 years. For a sensitivity of 0.90 the ratio of cases detected at organised screening that would have progressed to invasive cancer to those detected at opportunistic screening was 1.01. The corresponding figure for a sensitivity of 0.75 was 0.985, and for a sensitivity of 0.50 it was 0.984.

### Discussion

We used prospectively collected data to compare organised and opportunistic screening in terms of ability to detect cancer *in situ*. The study cohort comprised all women who had been screened at least once. The time period included the time of introduction of large scale screening, when prevalent cases predominated, and the more stationary phase thereafter. Since it is assumed premalignant lesions are eliminated by screening, we used cancer *in situ* rather than invasive cancer as the end point. Our results would not reflect the true impact of screening on the incidence of cervical cancer and its mortality if cancer *in situ* cases detected at organised and opportunistic screening differed in their probability of progressing to invasive cancer. The dynamic modelling suggested, however, that this difference, if any, was negligible. Hence, we propose the detection ratio as a valid measure of

**Table II** Results of fitting logistic regression models to the detection ratios of cervical cancer *in situ* in the county of Uppsala, Sweden, 1969–88

| *Model* | *Deviance* | *d.f.* | *Change in deviance* | *d.f.* | P-*value* |
|---|---|---|---|---|---|
| 1. General mean only | 1220.4 | 400 | 27.7 | 1 | <0.001 |
| 2. 1 + type | 1174.7 | 399 | 236.4 | 7 | <0.001 |
| 3. 2 + age | 938.2 | 392 | 365.6 | 3 | <0.001 |
| 4. 3 + time period | 572.6 | 389 | 118.2 | 6 | <0.001 |
| 5. 4 + elapsed time | 454.4 | 383 | 33.5 | 3 | <0.001 |
| 6. 5 + type × period | 420.9 | 380 | | | |

**Table III** Odds ratios for detection of cancer *in situ* of the cervix uteri, and their approximate 95% confidence intervals in cytological smears taken in the county of Uppsala, Sweden during 1969–88

| Factor | *Univariate models* Odds ratio | 95% CI | *Multivariate model* Odds ratio | 95% CI |
|---|---|---|---|---|
| Type of screening | | | | |
| Organised | 1.00 | Reference | | |
| Spontaneous | 0.69 | 0.61–0.79 | | |
| Type of screening[a] | | | | |
| 1969–73 | | | 2.19 | 1.75–2.73 |
| 1974–78 | | | 1.39 | 1.03–1.87 |
| 1979–83 | | | 0.89 | 0.66–1.21 |
| 1984–88 | | | 0.75 | 0.52–1.09 |
| Age at screening test (years) | | | | |
| ⩽24 | 0.47 | 0.38–0.58 | 0.42 | 0.34–0.52 |
| 25–29 | 1.00 | 0.84–1.19 | 0.91 | 0.76–1.08 |
| 30–34 | 1.00 | Reference | 1.00 | Reference |
| 35–39 | 0.87 | 0.72–1.05 | 0.95 | 0.79–1.15 |
| 40–44 | 0.52 | 0.41–0.66 | 0.55 | 0.44–0.70 |
| 45–49 | 0.53 | 0.41–0.68 | 0.49 | 0.38–0.62 |
| 50–54 | 0.19 | 0.12–0.30 | 0.19 | 0.12–0.29 |
| 55+ | 0.17 | 0.11–0.25 | 0.15 | 0.10–0.23 |
| Time period of test | | | | |
| 1969–73 | 2.25 | 1.93–2.62 | 1.67 | 1.21–2.30 |
| 1974–78 | 1.00 | Reference | 1.00 | Reference |
| 1979–83 | 0.65 | 0.55–0.77 | 1.03 | 0.71–1.50 |
| 1984–88 | 0.40 | 0.33–0.48 | 0.74 | 0.49–1.12 |
| Elapsed time since previous smear (years) | | | | |
| <1 | 1.05 | 0.79–1.39 | 0.81 | 0.60–1.10 |
| 1–2 | 0.44 | 0.32–0.59 | 0.37 | 0.27–0.51 |
| 2–3 | 0.43 | 0.31–0.60 | 0.35 | 0.25–0.50 |
| 3–4 | 0.72 | 0.51–1.03 | 0.59 | 0.41–0.84 |
| 4–5 | 0.95 | 0.66–1.38 | 0.80 | 0.55–1.16 |
| 5+ | 1.00 | Reference | 1.00 | Reference |
| First smear | 1.27 | 0.97–1.67 | 0.82 | 0.61–1.10 |

[a]For each time period organised screening was used as the reference category. The multivariate estimates are from model 6 in Table II. This model allows the effects of spontaneous screening to vary between time periods. Organised screening was used as the reference category.

screening efficiency for the purpose of comparing organised and opportunistic screening.

According to our data, opportunistic screening was as likely to detect cancer *in situ* as was organised screening. The overall similarity between the two types of screening is seen in Figures 3 and 4, but multivariate analysis is required for a more informative quantitative comparison. The crude odds ratio of 0.69 – indicating a 31% lower probability of detecting cancer *in situ* at opportunistic screening than at organised screening – is confounded by the difference in the distribution of the two screening types over time (Figure 1) and age (Figure 2).

After adjustment for time period and age it became evident that opportunistic screening was more efficient in detecting cancer *in situ* than was organised screening during the first 10 years of our study. However, during the last 10 years there was no significant difference between the two types; if any, there was a tendency for organised screening to be more efficient (Table III). These estimates were also adjusted for time interval between smears. To reflect reality we would probably prefer a model without adjustment for this variable. In fact, this gives similar odds ratios, but indicates a smaller advantage of opportunistic screening during the first 10 years and a somewhat larger advantage for organised screening in the last decade, although these estimates are still not statistically significant.

The interaction between type of screening and time period may be best understood in light of the decreasing overall detection ratio from the first to the last 5 year period (Table III). Several factors are likely to cause this trend. First, the diagnostic yield should be higher during the first years after screening was introduced since many prevalent lesions are detected at a first screening. Secondly, the diagnostic criteria for cancer *in situ* – including severe dysplasia – are vague, vary greatly between pathologists (Bergström *et al.* 1993) and have probably become more stringent in the county of Uppsala in recent years. Thirdly, it is probable that the opportunistic screening has changed from mainly smear-taking on indication to routine examination in maternity wards and family planning clinics. Theoretically, the negative trend in the detection ratio may also result from a decrease in test sensitivity or a reduction of the incidence of cancer *in situ* in the screened population. These factors are unlikely to play a practical role, however; if anything, there is evidence that the incidence of cancer *in situ* has increased, at least in younger women (Gustafsson, 1986).

We believe that subject selection is more important. Sexually active women at relatively high risk of developing cancer *in situ* may have been overrepresented among screened women during the early years. They may also have been detected more readily at opportunistic than at organised screening. This could account for the finding that the detection ratio was approximately twice as high at opportunistic as at organised screening during the first 5 year period but did not differ significantly in the last decade (Table III).

Ths risk of being diagnosed with cancer *in situ* increases for at least 4 years after a normal smear. The higher odds ratio at less than 1 year may be the result of misclassification of some symptomatic or secondary smears (Table III).

We failed to identify biases that could have concealed a true benefit of organised screening. Indeed, organised screening was partly a safety system for women considered to be at high risk, as described in the Materials and methods section. Nevertheless, more than 70% of all cancers *in situ* were detected at opportunistic screening. Although we have no empirical support, it is conceivable that the test sensitivity differs between organised and spontaneous screening. However, the empirical evidence suggests that midwives are at least as capable of taking smears as general practitioners (Ahlgren *et al.*, 1969; Bhargava *et al.*, 1993; Mitchell, 1993).

How representative are our data? Over the years, opportunistic screening has accounted for 75–80% of all smears in Sweden as a whole (National Board of Health and Welfare, 1982; Pettersson *et al.*, 1985) and for 78% of primary smears in the county of Uppsala. Screening for cervical cancer has been as successful in the county of Uppsala as in Sweden (National Board of Health and Welfare, 1973–91; Gustafsson and Adami, 1989); in the birth cohorts screened most extensively, the incidence of and mortality from cervical cancer have been reduced by about 70% in Sweden (Gustafsson and Adami, 1990). Hence our results can probably be generalised at least to the Swedish population.

Are our data informative for those intending to start cytological screening and for those who want to improve the efficiency of ongoing screening, organised or opportunistic? At least they challenge our prior belief in a definite advantage of organised screening, shared by many other investigators (Hakama, 1982, 1986; Draper and Cook, 1983; IARC, 1986; Läärä *et al.*, 1987; Anderson *et al.*, 1988; Hakama and Louhivuori, 1988; Day, 1989; Lynge *et al.*, 1989, 1992; Storm and Jensen, 1989; Koopmanschap *et al.*, 1990*a*,*b*). Although preference for one type of screening by certain groups of women could not be assessed directly in our observational study, we found no support for the idea that opportunistic screening selectively reaches women at low risk. Overscreening is a more evident feature of opportunistic screening in our setting. An increase in the mean interval between smears from less than 2 years to, say, 3 years or longer would probably reduce the costs substantially, whereas the benefit of screening would remain largely similar (Gustafsson and Adami, 1992). A longer interval between opportunistic screenings can probably be brought about through a combination of organisational change, a well-considered health care policy and public as well as professional education. We also believe that an important aspect of the Swedish setting is the fact that diagnostic work-up and treatment of abnormal smears are standardised and independent of type of screening. Therefore we regard it as important to make similar studies in other settings and to incorporate also the economic aspects of type of screening before designing a screening programme.

We wish to suggest a more optimistic view of the efficiency of opportunistic screening. Evidently, the claim that the successful control of cervical cancer in Sweden is attributable mainly to organised screening is not well founded. In many settings, a well-thought-out use of means of promoting smear-taking initiated by women, midwives or doctors might be an efficient way to stimulate screening in a large majority of women and to increase the benefit of activities already in progress.

**Acknowledgements**
This study was supported by grants from the Swedish Cancer Society.

### References

AHLGREN M, LINDBERG LG, NORDQVIST S AND STORMBY NG. (1969). Mass screenaing for cervical cancer with the aid of nurses and an administrative computer service. *Acta Obstet. Gynecol. Scand.*, **48** (Suppl. 3), 58–60.

ANDERSON GH, BOYES DA, BENEDET JL, LE RICHE JC, MATISIC JP, SUEN KC, WORTH AJ, MILLNER A AND BENNETT OM. (1988). Organisation and results of the cervical cytology screening programme in British Columbia, 1955–1985. *Br. Med. J.*, **296**, 975–978.

BERGSTRÖM R, ADAMI HO, GUSTAFSSON L, PONTÉN J AND SPARÉN P. (1993). Detection of preinvasive cancer of the cervix and the subsequent reduction in invasive cancer. *J. Natl Cancer Inst.*, **85**, 1050–1057.

BHARGAVA VL, VERMA K, SHARMA R, BATRA S, ANANDALAKSHMY. (1993). A hospital-based study on the use of paramedical personnel for clinical downstaging of cancer cervix. *Ind. J. Med. Res.* B, **98**, 65–68.

BRESLOW N. (1992). Use of the logistic and the related models in longitudinal studies of chronic disease risk. In *Statistical Models for Longitudinal Studies of Health, Monograph in Epidemiology and Biostatics*, Vol. 16, Dwyer JH, Feinleib M, Lippert P and Hoffmeister H. (eds) pp. 163–197. Oxford University Press: Oxford.

CHAMBERLAIN J. (1984). Failures of the cervical cytology screening programme (letter). *Br. Med. J.*, **289**, 853–854.

COOK GA AND DRAPER GJ. (1984). Trends in cervical cancer and carcinoma *in situ* in Great Britain. *Br. J. Cancer*, **50**, 367–375.

DAY NE. (1984). Effect of cervical cancer screening in Scandinavia. *Obstet. Gynecol.*, **63**, 714–718.

DAY NE. (1989). Screening for cancer of the cervix. *J. Epidemiol. Comm. Health*, **43**, 103–106.

DRAPER GJ AND COOK GA. (1983). Changing patterns of cervical cancer rates (letter). *Br. Med. J.*, **287**, 510–512.

ELWOOD JM, COTTON RE, JOHNSON J, JONES GM, CURNOW J AND BEAVER MW. (1984). Are patients with abnormal cervical smears adequately managed? *Br. Med. J.*, **289**, 891–894.

GUSTAFSSON L. (1986). The natural history of cancer of the cervix uteri. A simulation study based on Swedish statistics for 1958–1981. Teknikum, Uppsala University, UPTEC 8607R, Uppsala.

GUSTAFSSON L AND ADAMI HO. (1989). Natural history of cervical neoplasia: consistent results obtained by an identification technique. *Br. J. Cancer*, **60**, 132–141.

GUSTAFSSON L AND ADAMI HO. (1990). Cytologic screening for the uterine cervix in Sweden evaluated by identification and simulations. *Br. J. Cancer*, **61**, 903–908.

GUSTAFSSON L AND ADAMI HO. (1992). Optimization of cervical cancer screening. *Cancer Causes Control*, **vol 3**, 125–136.

GUSTAFSSON L, SPARÉN S, GUSTAFSSON M, PETTERSSON B, WILANDER E, BERGSTRÖM R AND ADAMI HO. (1995). Low efficiency of cytologic screening for cancer in situ of the cervix in older women (submitted).

HAKAMA M. (1982). Trends in the incidence of cervical cancer in the Nordic countries. In *Trends in Cancer Incidence – Causes and Practical Implications*. Knut M. (ed.) pp. 279–292. Hemisphere: New York.

HAKAMA M. (1986). Efficacy of screening for cervical cancer. *Banbury Report*, **21**, 45–54.

HAKAMA M AND LOUHIVUORI K. (1988). A screening programme for cervical cancer that worked. *Cancer Surveys*, **7**, 403–416.

HILL GB AND ADELSTEIN AM. (1967). Cohort mortality from carcinoma of the cervix. *Lancet*, Sept 16, 605–606.

IARC WORKING GROUP ON EVALUATION OF CERVICAL CANCER SCREENING PROGRAMMES. (1986). Screening for squamous cervical cancer: duration of low risk after negative results of cervical cytology and its implication for screening policies. *Br. Med. J.*, **293**, 659–664.

KNOX EG AND WOODMAN CBJ. (1988). Effectiveness of cancer control programme. *Cancer Surveys*, **7**, 379–401.

KOOPMANSCHAP MA, VAN OOTMARSSEN GJ, VAN AGT HMA, VAN BALLEGOOIJEN M, HABBEMA JDF AND LUBBE KTN. (1990*a*). Cervical cancer screening: attendance and cost-effectiveness. *Int. J. Cancer*, **45**, 410–415.

KOOPMANSCHAP MA, LUBBE KTN, VAN OORTMARSSEN GJ, VAN AGT HMA, VAN BALLEGOOIJEN M AND HABBEMA JDF. (1990*b*). Economic aspects of cervical cancer screening. *Soc. Sci. Med.*, **30**, 1081–1087.

LYNGE E, MADSEN M AND ENGHOLM G. (1989). Effect or organized screening on incidence and mortality of cervical cancer in Denmark. *Cancer Res.*, **49**, 2157–2160.

LYNGE E, ENGHOLM G AND MADSEN M. (1992). Organiseret screenings betydning for udviklingen af livmoderhalskraeft i Danmark i 1968–87, Ugeskr. Laeger **154/19**, 1330–1334.

LÄÄRÄ E, DAY NE AND HAKAMA M. (1987). Trends in mortality from cervical cancer in the Nordic countries: association with organized screening programmes. *Lancet*, **30**, 1247–1249.

MEDICINALVÄSENDET. (1968). Samling av författningar och cirkulär m.m. **Nr 1**, Kungl. Boktr.: Stockholm. (in Swedish).

MILLER AB, ANDERSON MB, BRISSON J, LAIDLAW J, LE PITRE N, MALCOLMSON P, MIRWALDT P, STUART G AND SULLIVAN W. (1991*a*). Report on national workshop on screening for cancer of the cervix. Special supplement, *Can. Med. Assoc. J.*, **145**, 1301–1325.

MILLER AB, CHAMBERLAIN I, DAY NE, HAKAMA M AND PROROK PC. (eds). (1991*b*). *Cancer Screening, UICC Project on Evaluation of Screening for Cancer*, pp. 199–201. Cambridge University Press: Cambridge.

MITCHELL H. (1993). Pap smear collected by nurse practitioners: a comparison with smears collected by medical practitioners. *Oncol. Nurse Forum*, **20**, 807–810.

MURPHY MFG, CAMPBELL MJ AND GOLDBLATT PO. (1987). Twenty years' screening for cancer of the uterine cervix in Great Britain, 1964–84: further evidence for its ineffectiveness. *J. Epidem. Commun. Health*, **42**, 49–53.

NATIONAL BOARD OF HEALTH AND WELFARE. (1973–91). *Principles and Routines for Gynecological Health Examinations*. Report from group of experts of National Board of Health and Welfare. (In Swedish). Pm 1/82 Stockholm.

NATIONAL BOARD OF HEALTH AND WELFARE. (1973–91). *Cancer Incidence in Sweden*, annual publications 1969–88. The Cancer Registry: Stockholm.

NHS CERVICAL SCREENING PROGRAMME. (1991). *First Annual Report of the NHSCSP*. Hall: Oxford.

PAPANICOLAOU GN. (1954). *Atlas of Exfoliative Cytology*. Commonwealth Fund, Harvard University Press: Cambridge, MA.

PAPANICOLAOU GN AND TRAUT HF. (1943). *Diagnosis of Uterine Cancer by the Vaginal Smear*. Commonwealth Fund: New York.

PAPANICOLAOU GN, TRAUT HF AND MARCHETTI AA. (1948). *The Epithelia of Woman's reproductive Organs. A Correlative Study of Cyclic Changes*. Commonwealth Fund: New York.

PARKIN DM, NGUYEN-DINH X AND DAY NE. (1985). The impact of screening on the incidence of cervical cancer in England and Wales. *Br. J. Obstet Gynaecol.*, **92**, 150–157.

PETTERSSON F, BJÖRKHOLM E AND NÄSLUND I. (1985). Evaluation of screening for cervical cancer in Sweden: trends in incidence and mortality 1958–80. *Int. J. Epidemiol.*, **14**, 521–527.

SOSFS. (1982). (SOCIALSTYRELSENS FÖRFATTNINGSSAMLING) (M) 1982:69. Nordstedts Tryckeri: Stockholm.

SOSFS. (1984). (SOCIALSTYRELSENS FÖRFATTNINGSSAMLING) (M) 1984:32. Nordstedts Tryckeri: Stockholm.

STORM HH AND JENSENH OM. (1989). Developing effective policy for the prevention of cancers. In *Reducing the Risk of Cancer*, Heller T, Davey B and Bailey L. (eds) pp. 165–170. Hodder & Stoughton: London.